# Impact of intravesical hyaluronic acid treatment on bladder inflammation in interstitial cystitis rat model

**DOI:** 10.1590/S1677-5538.IBJU.2017.0713

**Published:** 2018

**Authors:** Ilker Fatih Sahiner, Hakan Soylu, Erhan Ates, Nuray Acar, Ismail Ustunel, Ahmet Danısman

**Affiliations:** 1 Department of Urology, Akdeniz University School of Medicine, Antalya, Turkey; 2 Department of Histology and Embryology, Akdeniz University School of Medicine, Antalya, Turkey; 3 Department of Urology, Adnan Menderes University School of Medicine, Aydin, Turkey

**Keywords:** Interstitial cystitis, Inflammation, Hyaluronic Acid

## Abstract

**Objective:**

To evaluate the effect of intravesical hyaluronic acid (HA) treatment on inflammatory cells and the severity of inflammation in an interstitial cystitis rat model created with hydrogen chloride (HCL) via immunohistochemical studies and myeloperoxidase activity for the first time in the literature.

**Materials and Methods:**

A total of 30 adult female white Rattus Norvegicus rats were divided into 3 groups as the HCL group, hyaluronic acid treatment (HCL-HA) group and control group. Chemical cystitis was created by administering HCL(400 microL,10 mM) except control group. A single dose of intravesical HA(0.5 mL,0.8 mg/mL) was administered to the treatment group. The bladder tissues of all subjects were immunohistochemically stained. The cell surface markers were used to evaluate inflammatory cell infiltration. Mast cell activation and IL-6 was evaluated to assess the inflammation and severity of inflammation, respectively. Myeloperoxidase activity was measured as it shows neutrophil density. Statistical significance was accepted as P<0.05.

**Results:**

It was observed that there was rich monocyte, T lymphocyte, B lymphocyte, and Natural Killer cells infiltration and high IL-6 levels in the bladder tissue after the intravesical hydrogen chloride instillation, especially in the stroma layer(p<0.005). In the HCL-HA group, severity of inflammation had statistically significantly regressed to the levels of the control group(p<0.005). An increase was observed in the bladder myeloperoxidase activity of the HCL group compared to the other two groups(p<0.05).

**Conclusions:**

Single dose intravesical hyluronic acid instillation reduces inflammatory cell infiltration and the severity of bladder inflammation in the rat model of bladder pain syndrome/interstitial cystitis.

## INTRODUCTION

The European Society for the Study of Interstitial Cystitis (ESSIC) and the European Association of Urology (EAU) define Bladder Pain Syndrome (BPS)/Interstitial Cystitis (IC) as the complaint of suprapubic pain associated with filling of the bladder accompanied by other symptoms such as increased frequency of daytime and nighttime urination, in the absence of a urinary infection or other pathology (1, 2).

The etiology of BPS/IC is still not fully understood. Causes of urothelial dysfunction such as glycosaminoglycan (GAG) layer disorders and inhibition of urothelial cell proliferation have been reported as etiological factor (3, 4). Intravesical hyaluronic acid (HA), a treatment option recommended by guidelines, strengthens the urine-tissue barrier by integrating with the GAG layer and creates an anti-inflammatory effect by inhibiting the leukocyte migration, adhesion of immune complexes, and bonding with specific receptors (I-CAM 1) that occur during the inflammatory process (5, 6).

Lymphocytes and other cells express a large number of different marker molecules that can be used to identify cells and characterize the cell type on their surfaces. These marker molecules were named CD (Cluster of Differentiation) by the International Human Leukocyte Differentiation Antigens (HLDA) Study Group (7). IL-6 is a pro-inflammatory cytokine produced by a variety of cell types including endothelial cells, macrophages, fibroblasts, and mast cells and indicates the severity of inflammation (8). Studies revealed that mast cells have a critical role on many inflammatory diseases. Increases in mucosal damage and activated mast cells in biopsies of patients with IC have been shown (9). Also, mast cells have been shown to be active in bladder injury models generated by intravesical instillation of protamine sulfate (10).

Myeloperoxidase is an enzyme found in neutrophil granules. A direct correlation has been shown to exist between the measurement of MPO activity in tissue samples and neutrophil counts (11).

Histopathologically, it has been shown that there is primarily an inflammatory cellular infiltration limited to the lamina propria which consists of lymphocytes and plasma cells in IC cases (12). Studies on animal models of IC have revealed neutrophil infiltration and activation of some inflammatory cytokines in the bladder (13).

In our study, we tried to show the effects of treatment on the rat model of IC treated with intravesical single dose HA with myeloperoxidase activity and by making an immunohistochemical assessment with polyclonal antibodies.

## MATERIALS AND METHODS

### The Animal Model

Following approval from the animal ethics committee of the Akdeniz University (2013.10.02), 30 adult female white Rattus norvegicus rats (weight 200-250 gr) were selected as subjects. The rats were divided into 3 groups including the treatment group (HA-treated group) in which chemical cystitis was created by administering hydrogen chloride (HCl) and then HA was administered, the disease group (HCl group) in which chemical cystitis was created using HCl but no treatment was administered, and the control group (PBS group) in which the stress of the test subjects was mimicked with phosphate buffered saline (PBS), so that there would be 10 rats in each group. After administering intraperitoneal anesthesia (ketamine hydrochloride 30 mg/kg and xylazine hydrochloride 100 mg/ kg) to the subjects in the HA-treated group and HCl group, a sterile 1.3 mm feline catheter was implanted transurethrally. The urine in the bladder was aspirated. Afterwards, 400 microL of 37% fuming HCl (Sigma-Aldrich, St. Louis, MO, USA) was diluted so as to be 10 mM. The prepared HCl was transurethrally instilled intravesically in the subjects in the HCl HA-treated group and HCl group and was left to wait for 10 minutes. The acid was then aspirated from the bladder and removed. For bladder neutralization, the bladder was first washed with 8.4% M sodium bicarbonate followed by 0.9% NaCl solution. At this stage, the subjects in the PBS group were transurethrally catheterized and 400 microL PBS was intravesically administered to their bladders so as to mimic the stress of the subjects in the HCl HA-treated group and the HCl group. This applied solution was left to wait for 10 minutes in the bladder and then removed by aspiration. After HCI instillation, the subjects were left to wait in the laboratory overnight for chemical cystitis to form and for the inflammatory response to develop. The following day, the bladders of the subjects in all groups were catheterized and the urine in the bladder aspirated, after which a single session of 0.5 mL, 0.8 mg/mL HA (Hyacyst, Syner-Med, Surrey, UK) was administered to the HA-treated group and a PBS solution was intravesically instilled in the HCl group and PBS group. After waiting for 20 min, the bladder was emptied and the subjects were followed in the laboratory until the sacrification procedure. Two days after treatment and the placebo procedure, irreversible anesthesia was given to the subjects with 50 mg/kg ketamine. Then, all the subjects were sacrificed and their bladders were evacuated with a lower abdominal midline incision. Some of the bladder samples obtained were embedded in paraffin and placed in 4% formalin. The others were then stored in liquid nitrogen to perform a myeloperoxidase assay.

## HISTOPATHOLOGICAL ASSESSMENT

### Immunohistochemical analysis

We used CD (Cluster of Differentiation) system to detect cell surface markers in the area of inflammation in our study. CD3 was used as a T lymphocyte surface marker, CD 14 as a monocyte surface marker, CD 19 as a B lymphocyte surface marker, and CD56 as a natural killer surface marker. We used IL-6 to assess the severity of inflammation.

The samples which were set in 4% formaldehyde for about 12 hours were then washed in tap water for 2 hours and dehydrated in ascending alcohol series. They were then made transparent in xylol and embedded in paraffin. In order to make routine light microscopic observations and to perform an immunohistochemical technique, sections with a thickness of 5 μm were placed on polylysine-coated slides. After paraffin removal, the sections were boiled for 7 min (4 min + 3 min) in citrate buffer (pH: 6.0) for antigen retrieval and allowed to cool at room temperature for 20 min. The sections were then left to wait for 20 minutes in 3% hydrogen peroxide to block endogenous peroxidase activity. After this procedure, Ultra V block (Lab-Vision, Fremont, CA, USA) was applied to the sections for 7 min in a humidified chamber at room temperature. After removal of excess serum, sections were incubated overnight in a humidified chamber at 4°C with primary antibodies: CD3 rabbit polyclonal antibody (Bioss Biotechnology; bS-0765R) in a 1:500 dilution; CD 14 rabbit polyclonal antibody (Bioss Biotechnology; bs-1192R) in a 1:500 dilution; CD19 rabbit polyclonal antibody (Bioss Biotechnology; bs-4755R) in a 1:400 dilution; CD 56 rabbit polyclonal antibody (Bioss Biotechnology; bS-0805R) in a 1:500 dilution; and IL-6 rabbit polyclonal antibody (Bioss Biotechnology; bs-4540R) in a 1:500 dilution. The following day, the sections were washed 3 times with PBS solution for 5 minutes and then incubated for 30 minutes at room temperature in a humidified chamber with a biotin marked secondary antibody; anti-rabbit IgG (BA-1000) in a 1:500 dilution. The slides were washed 3 times for 5 min with PBS and the emissions obtained were developed with diaminobenzidine (K3466; Dako). The sections were counterstained with hematoxylin, dehydrated in ascending alcohol series, and examined under a light microscope (Zeiss, Oberkochen, Germany) after closing them with Kaiser's glycerin gelatine (Merck; OB514196, NJ, USA).[Table t1]

### Semi-quantitative evaluation

The immunoreactive cells positively stained with the markers studied in all groups and their immunostaining densities were semi-quantitatively assessed by two observers with the method mentioned below.

**Table t1:** 

No staining	=	Grade 0
Cellular staining between 0-25%	=	Grade 1
Cellular staining between 25%- 50%	=	Grade 2
Cellular staining between 50%- 75%	=	Grade 3
Cellular staining between 75%- 100%	=	Grade 4

### Detection and count of mast cell

Toluidine blue powder (0.5 g) (Toluidine Blue O, Merck, 115930) was dissolved in 100 mL distilled water. Formalin fixed paraffin embedded samples were cut into 5 pm sections and placed on slides. After deparaffinization, sections were taken to water and were stained with this solution for 1 min. They were rinsed in water for 3-5 min, differentiated respectively in 95% alcohol, absolute alcohol, and were cleared. Finally, the sections were taken through graded alcohols to xylene and mounted in entellan. Sections were examined by light microscopy (Zeiss, Oberkochen, Germany). Photographs of PBS, HCL and HCL+HA groups were taken with an Axioplan^®^ microscope (Zeiss, Oberkochen, Germany) and mast cells were counted through the use of the Image J (http://imagej.nih.gov/ij/) program.

### Measuring of Myeloperoxidase activity

Because the measurement of myeloperoxidase in the environment indirectly indicates neutrophil concentration, MPO was used as a neutrophil marker in our study.

The other half of the collected tissues was stored in a nitrogen tank at −196°C. The Myeloperoxidase Activity Assay Kit (Abcam, ab111749) was used to detect neutrophil accumulation and activity in tissues. The tissues were removed from the nitrogen tank and placed in 400 μL of assay buffer to be homogenized with a sonicator and were then centrifuged for 10 minutes at 14000 g. The supernatants were taken into Eppendorfs and 50 μL of each supernatant was added to the 96-well microwell plate. 50 μL of the positive control was added to one microwell and fluorescein standard was added so as to be 0, 10, 20, 30, 40, and 50 pmoL/microwell in it in the given order in 6 microwells so as to form a standard curve. Five minutes after the standard microwells were stirred, they were scanned at 485/525 nm by an ELISA Reader. Finally, 46 μL of assay buffer, 2 μL of myeloperoxidase substrate solution, and 2 μL of myeloperoxidase probe were added to the other microwells except for the standards to start the reaction and these were scanned at 485/525 nm. After this scanning, the plate was incubated in a dark place for 30 minutes and re-scanned at 485/525 nm to determine the MPO concentrations of the samples.

### Statistical analysis

The immunohistochemistry datas obtained from Image J analysis were compared with Student's t-test. Comparisons were made among PBS, hydrochloric acid and hydrochloric acid+hyaluronic acid structures. Probability values of less than 0.05 were considered significant. All statistical analyses were performed using Sigma Stat 3.5 (Statcon, Witzenhausen, Germany).

## RESULTS

### Immunohistochemical and Semi-quantitative Evaluation Results

**Figure 1:**
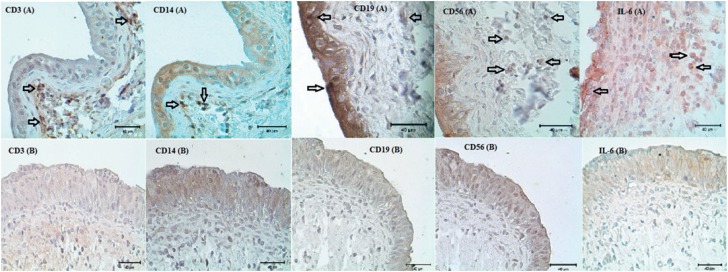
Images representing immunohistochemical staining of CD3, CD14, CD19, CD56 and IL-6 in HCl group (A) and HCl-HA group (B). Black arrows indicate stained inflammatory cells. It is observed that inflammatory cell infiltration is more common in stroma and smooth muscle.

**Figure 2:**
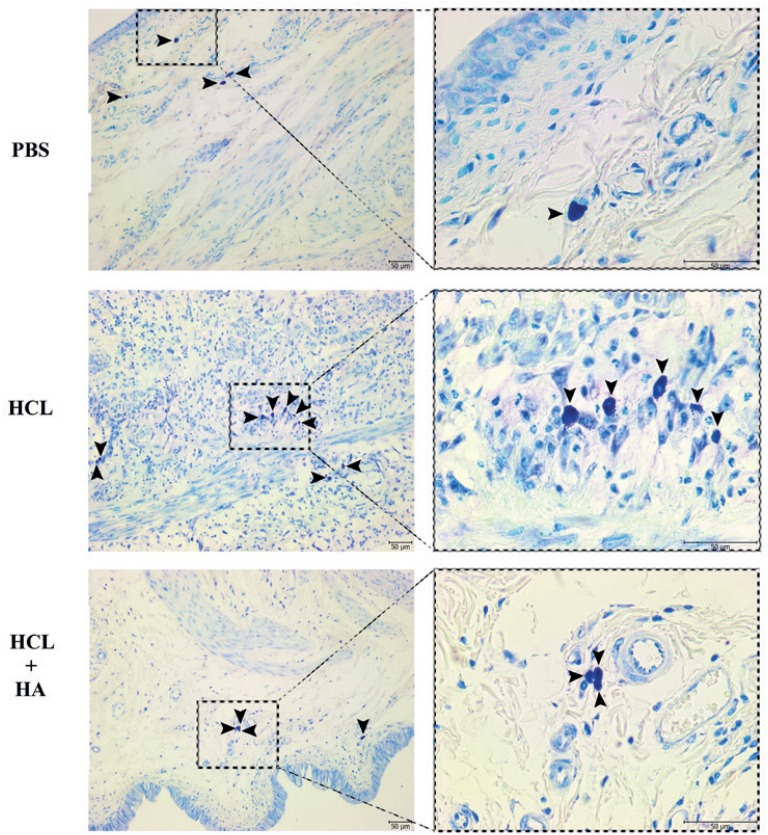
Image representing mast cell count between three groups. Arrowhead shows mast cells.

**Figure 3:**
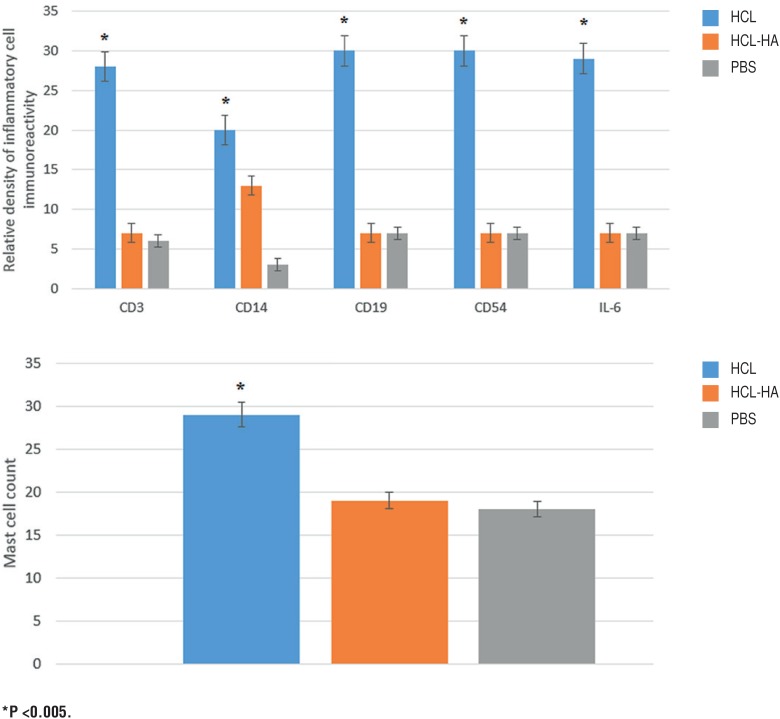
Relative density of CD3, CD14, CD19, CD56 and IL-6 immunoreactivity. Inflammatory response was found to regress to the control group (PBS) levels significantly compared with Hcl group after intravesical HA treatment. The number of activated mast cells is significantly reduced after intravesical HA treatment. *P <0.005.

The immunohistochemical assessment sections were semi-quantitatively evaluated by two different observers. After immunohistochemical staining, it was observed that there was infiltration rich in monocyte cells and very rich in T lymphocyte, B lymphocyte, and natural killer cells in the bladder tissue of the HCl group, especially in the stroma layer. Levels of IL-6 correlated with the severity of inflammation were elevated to peak levels in the HCl group, especially in the stroma and smooth muscle layer. In the HA-treated group which was treated with single dose intravesical HA, it was observed that the rich infiltration formed in the stroma layer of the bladder by inflammation cells and the severity of inflammation had statistically significance regressed to the levels of the PBS group (p<0.005) (Figure-1). Likewise, the increase in the number of activated mast cells in the HCL group significantly declined to level of the PBS group after intravesical HA treatment (p<0.005) (Figure-2). In light of these findings, it has been determined that 0.5 mL,0.8 mg/mL of intravesical HA instillation administered to the subjects in the treatment of chemical cystitis statistically significantly suppresses the inflammatory response (p<0.005) (Figure-3).

### Myeloperoxidase Assay Results

**Figure 4:**
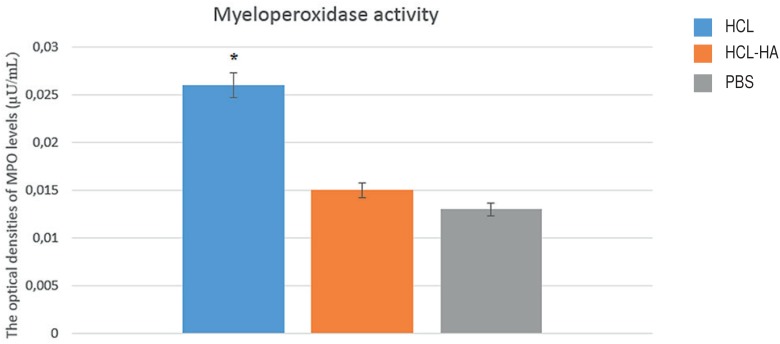
Image representing MPO activity increase HCl group *P <0.05.

An increase was observed in bladder MPO activity in the HCl group compared to the HA-treated and PBS group (p<0.05). MPO activity levels in the HA-treated and PBS groups were found to be close to each other (Figure-4). It was observed that a result close to the pre-disease condition was achieved with HA treatment.

## DISCUSSION

BPS/IC is a clinical diagnosis based on symptoms of chronic pain originating in the bladder, with widespread prevalence. The uncertainty of its etiology causes difficulties in treatment. There are various theories on its pathophysiology. It has been hypothesized that IC/BPS could be pathophysiologically related to a defect of the GAG layer of the bladder mucosa (4). The relevant components of the GAG layer include HA, heparin sulfate, chondroitin sulfate (CS), dermatan sulfate, and keratin sulfate (14). Among the many reported functions of the surface GAG layer are the prevention of surface entrapment of substances and the regulation of transepithelial molecule movements. These polysaccharides containing negatively charged sulfate form a thin layer in the form of a physical barrier between the cell surface and urine because they are highly hydrophilic (15). Parsons has shown that the GAG layer is significantly reduced in BPS/IC patients and that the normal permeability barrier is thereby impaired (16). Subsequent animal experiments have shown that destruction of the GAG layer of urine can lead to bladder inflammation and hyperactivity (17).

The fact that urologists are prone to drug administration into the bladder, the inability to achieve the desired level of response from oral treatments, and the belief that this disease is based on impairment of the bladder mucosa have brought up the topic of intravesical treatments. The advantages of intravesical therapy are that it can provide more intensive use of therapeutic agents in the bladder and limit systemic side effects while being invasive and carrying the risk of infection are its disadvantages. Many agents have been investigated in BPS/IC treatment with the purpose of intravesical use. When heparin, one of the intravesical agents that support the GAG layer, was intravesically administered at 25000 IU in 5 cc sterile water, improvements were observed in 72% of patients (18). In a non-randomized study with chondroitin sulfate, another agent that supports the GAG layer, approximately 70% improvement in symptom scores was observed (19). In the study by Kallestrup et al. (20) on HA, another GAG supporting agent which is accepted for its long-term efficacy, improvement in the symptoms was observed in 65% percent of female patients who underwent instillation treatment once weekly during the first month and once monthly afterwards, and full cure was achieved after 3 years in 50% of patients. Another study involving 48 patients is also noteworthy due to demonstrating that HA treatment is also urodynamically effective and having a long follow-up period of 5 years (21). The formula for increasing the amount of HA by administering it from the outside can restore the integrity of the GAG layer by strengthening the barrier function. However, there is no objective assessment tool other than some surveys to assess the effectiveness of GAG replacement therapy (22). To the best of our knowledge, there are no other studies in the literature which demonstrate the results of HA treatment by assessing inflammatory cells in the tissue via immunohistochemical studies and MPO activity. In this respect, our study carries the distinction of being the first to do so.

Until recently, the only clinical material could be used in IC research and animal models has been created with the advent of experimental studies. It has been shown that when chemical cystitis models which have been created were compared in terms of both leukocyte counts and mast cell counts, they were found to be similar to IC and that therefore an animal model of IC can be created and studies can be done in this way. The IC model can be created with protamine sulfate (PS), cyclophosphamide, HCl, acetic acid, lipopolysaccharide, and uroplakin (23). In our study, we used HCl to create IC and pathological similarity with IC was found to be consistent with the literature.

Studies on animal models of IC have pointed at neutrophil infiltration, activation of certain inflammatory cytokines in the bladder, and increased expression of inflammatory genes as the source of symptoms (13, 24). Lv et al. (25) assessed results after intravesical HA in a PS-induced rat model of chemical cystitis with the immunohistochemical method and looked at IL-6 levels for the severity of inflammation. IL-6 levels which correlate with the severity of inflammation reached peak levels in the chemical cystitis induced group, while a significant drop was observed in the IL-6 levels of the group which was treated with single dose HA after chemical cystitis. We also found a significant decrease in mast cell count and IL-6 levels in our study with single dose intravesical HA treatment compared to the disease group and obtained a result consistent with the literature. In addition, the MPO activity level and the other immunohistochemical markers, namely CD3, CD14, CD19, and CD56, were found to have reached the level of control group rats in the group receiving HA treatment after the procedure and near normal mucosa, intact epithelium, and basal membrane layer presence were observed.

The limitation of our study included small number of experiment animals, evaluation of efficacy of single dose treatment and lack of histopathological results of long-term treatment. Long-term symptomatic remission with intravesical hyaluronic acid treatment has been shown in IC (26). Future works should investigate the long-term effects of intravesical hyaluronic acid treatment on bladder inflammation in patients with IC.

## CONCLUSIONS

Our study has shown that single dose intravesical HA instillation in the rat model of IC is effective in treatment by reducing inflammatory cell infiltration and the severity of inflammation, preserves the mucosal integrity of the bladder, and thus achieves histopathological improvement. The low cost and the lack of side effects may enable HA to be among the first drugs to be preferred for the treatment of IC. However, extensive research is needed to determine its functional effects on bladder capacity and compliance, and to determine to which extent its combined use with other methods enabling GAG layer repair will affect the topical effects of HA that we have identified.
